# ADAMTS-13 R1206K variant exhibits an open conformation and an enhanced proteolytic activity toward von Willebrand factor

**DOI:** 10.1016/j.jtha.2026.03.010

**Published:** 2026-03-25

**Authors:** Szumam Liu, Xiaxin Dong, Quintijn Bonnez, Zhirong Chai, Chan Meng, Madison Gil, Karen Vanhoorelbeke, Konstantine Halkidis, X. Long Zheng

**Affiliations:** 1 Department of Pathology and Laboratory Medicine, The University of Kansas Medical Center, Kansas City, Kansas, USA; 2 Institute of Reproductive and Developmental Sciences, The University of Kansas Medical Center, Kansas City, Kansas, USA; 3 Laboratory for Thrombosis Research, Kortrijk, Belgium; 4 Department of Internal Medicine, The University of Kansas Medical Center, Kansas City, Kansas, USA

**Keywords:** ADAMTS13, thrombosis, treatment, thrombotic thrombocytopenic purpura, von Willebrand factor, arginine methylation

## Abstract

**Background:**

Thrombotic thrombocytopenic purpura (TTP) is a life-threatening thrombotic microangiopathy caused by severe deficiency of ADAMTS-13 (a disintegrin and metalloprotease with thrombospondin type 1 repeats, 13), a plasma metalloprotease that cleaves von Willebrand factor (VWF). Recombinant ADAMTS-13 has been developed for treatment of TTP, but it requires a high dose and frequent administrations to achieve therapeutic efficacy.

**Objectives:**

The present study aims to identify more efficacious ADAMTS-13 variants for potential management of TTP.

**Methods:**

Site-directed mutagenesis, recombinant protein production and purification, enzyme kinetic and binding assays, and microfluidic thrombus formation were all used in the study.

**Results:**

ADAMTS-13 R1206K variant exhibits a significantly increased activity compared with wild-type ADAMTS-13 for proteolytic cleavage of multimeric VWF and its surrogate peptide. Additionally, the R1206K is significantly more efficacious for inhibiting platelet adhesion and aggregation on a collagen-coated surface under arterial flow. Biolayer interferometry assay reveals a faster association kinetics (*k*_a_) between R1206K and VWF73^L1603A^ substrate but a reduced binding affinity between R1206K and autoantibodies against ADAMTS-13, derived from immune-mediated TTP. Consistent with these results, the fluorescent energy transfers (FRETS)–VWF73 assay reveals a higher IC_50_ for autoantibodies (IgG4 4–16 and 4–20) to inhibit the R1206K than wild-type ADAMTS-13.

**Conclusion:**

These results demonstrate that ADAMTS-13 R1206K variant has an enhanced enzymatic activity, resulting from the disruption of autoinhibition that leads to an open conformation, which increases its substrate binding but reduces its autoantibody inhibition. This gain-of-function ADAMTS-13 variant may be further explored for the management of TTP and perhaps other inflammatory thrombotic disorders.

## 1 | INTRODUCTION

Thrombotic thrombocytopenic purpura (TTP) is a rare but life-threatening blood disorder, characterized by widespread formation of microvascular thrombosis. TTP is primarily caused by severe deficiency of ADAMTS-13 (a disintegrin and metalloprotease with thrombospondin type 1 repeats, 13) activity [1]. The only known biological function of ADAMTS-13 to date is to cleave ultralarge von Willebrand factor (VWF) multimers, which prevents the excessive platelet adhesion and aggregation, as well as thrombus formation at the sites of vascular injury [2]. Deficiency of plasma ADAMTS-13 activity leads to disseminated thrombosis in small arterioles and capillaries, the characteristic feature of TTP.

Patients with TTP usually present with severe thrombocytopenia, microangiopathic hemolytic anemia, and various degrees of organ damage [3]. There are at least 2 major types of TTP: congenital or hereditary TTP (hTTP), caused by mutations of *ADAMTS-13* [4–6], resulting in a reduced enzyme production or activity; and acquired or immune-mediated TTP (iTTP), resulting from the formation of autoantibodies that inhibit ADAMTS-13 activity [7,8] and/or accelerate ADAMTS-13 clearance from circulation [9,10].

Current therapy for hTTP primarily focuses on the replacement of missing ADAMTS-13 enzyme through frequent plasma infusion [11,12] or an administration of recombinant ADAMTS-13 [13]. The therapies for iTTP include daily therapeutic plasma exchange (TPE), caplacizumab, and immunosuppression, known as the triple therapy [14,15]. While these treatments have been highly effective, resulting in significantly reduced TTP mortality and morbidities, they still have many limitations. For instance, frequent plasma infusion carries a potential risk for infection or allergic/anaphylactic reaction and is inconvenient for patients to travel to infusion center. Unfortunately, plasma infusion alone does not appear to provide sufficient amount of ADAMTS-13 to reduce the long-term complications associated with TTP [16,17]. Recombinant ADAMTS-13 is more efficacious than plasma infusion, but it requires a high dose and frequent infusion to achieve desired therapeutic effects [18]. In some countries, this treatment may be cost prohibitive. TPE is highly efficacious, but it is an invasive procedure, requiring complex apheresis equipment and trained medical staff to perform [3,19]. Caplacizumab, an anti-VWF nanobody, which is added to TPE and immunosuppression, has been shown to accelerate platelet count recovery and reduce TTP exacerbation and relapse, but it may cause unwanted bleeding complications [20,21]. Immunosuppressives such as corticosteroids and rituximab that aim to reduce autoantibody production may be associated with an increased risk of infections [22,23]. Thus, there are still unmet needs for developing better, cost-effective, and more effective therapies for TTP.

Building on our recent work published [24], we identified an ADAMTS-13 variant with a change of arginine to lysine at position of 1206 in the CUB1 domain. This change appears to disrupt autoinhibition of the distal domains, resulting in an opened conformation of ADAMTS-13. This R1206K variant exhibits an enhanced enzymatic activity but reduced susceptibility to autoantibody inhibition. This novel gain-of-function ADAMTS-13 variant may be explored for development as a next-generation therapy for TTP and perhaps other inflammatory and thrombotic disorders.

## 2 | METHODS

### 2.1 | Expression, purification, and quantification of recombinant ADAMTS-13

Human recombinant wild-type (WT)-ADAMTS-13 and R1206K variant were expressed in stably transfected HEK293 cells as previously described [25,26]. For kinetic analyses, the C-terminal His-tagged recombinant proteins were purified from serum-free conditioned medium using nickel-nitrilotriacetic acid affinity chromatography (Qiagen). Following elution, the proteins were dialyzed into a storage buffer (20 mM HEPES, 150 mM NaCl, pH 7.4). Protein concentration was determined by measuring absorbance at 280 nm and verified by Coomassie blue staining and western blotting using a monoclonal anti-His antibody. For qualitative multimer cleavage assays under denaturing conditions, ADAMTS-13 proteins in the concentrated conditioned media were utilized. In these instances, recombinant ADAMTS-13 antigen levels were quantified by an enzyme-linked immunosorbent assay (ELISA) (R&D system).

### 2.2 | Cleavage of VWF73 by ADAMTS-13 under different pH conditions

The proteolytic activity of human recombinant ADAMTS-13 and R1206K was assessed using the FRET-rVWF73 assay as previously described [27,28]. The reaction system contained 5 mM Bis-Tris, 25 mM CaCl_2_, 0.005% Tween-20, and 1 mM Pefabloc (Sigma-Aldrich) at pH 6 and pH 7, respectively. Murine plasma ADAMTS-13 activity was determined by a cattle FRET-rVWF71 substrate (kindly provided by Dr Josua Muia at Versiti, Milwaukee, Wisconsin) [29]. This assay was performed in a buffer consisting of 50 mM HEPES, 150 mM NaCl, 10 mM CaCl_2_, 0.05% Tween-20, and 1 mg/mL of bovine serum albumin (BSA) [30]. All reactions were performed in a 96-well opaque white Nunc plate (ThermoFisher) at 25 °C, and the rate of fluorescence generation was detected using a SpectraMax Gemini XPS plate reader (Molecular Devices).

### 2.3 | Biolayer interferometry

Binding interactions between WT-ADAMTS-13 or R1206K and an uncleavable VWF73 mutant (VWF73^L1603A^) were analyzed using biolayer interferometry (BLI) on the BLItz platform [31]. All experiments were performed at 25 °C in a Blitz buffer (phosphate-buffered saline containing 0.1% BSA and 0.02% Tween-20). For VWF73 binding assays, nickel-nitrilotriacetic acid biosensors were hydrated in a Blitz buffer for 100 seconds prior to protein loading. WT-ADAMTS-13 or R1206K was immobilized onto the biosensors by incubation for 300 seconds, followed by a 200-second wash in fresh Blitz buffer to remove unbound protein. The association phase was initiated by exposing the sensor-bound enzyme to serial concentrations of substrate VWF73^L1603A^ (0–18 μM) for 600 seconds, and dissociation was monitored for 1200 seconds in the Blitz buffer. In addition, immunoglobulin (Ig)G binding kinetics were determined using an antihuman IgG Fc capture biosensors preimmobilized with 20 nM of each human monoclonal IgG4 (3–3, 4–16, 4–20, and 4–41). After antibody capture, the sensors were exposed to increasing concentrations of WT-ADAMTS-13 or R1206K (0.125–80 nM) to record real-time association and dissociation. Binding signals were recorded as wavelength shifts (nanometers) over time (seconds), and kinetic parameters—association (*k*_on_), dissociation (*k*_off_), and equilibrium dissociation (*K*_d_)—were calculated using the Octet BLI Pro software (Gottingen) with a 1:1 global binding model. All experiments were performed in triplicate, and curve fitting was accepted only for *R*^2^ of ≥ 0.96 to ensure data reliability.

### 2.4 | Cleavage of multimeric VWF by ADAMTS-13 under denaturing conditions

Purified plasma VWF (150 nM) was incubated with WT-ADAMTS-13 or R1206K in various conditioned media at 37 °C for 4 hours on a dialysis membrane (0.25-μm pore size), floating over 50-mL dialysis buffer (10 mM Tris-HCl, pH 8.0, containing 1.5 M urea) in a conical tube [32,33]. The digested material was collected from the top of the membrane and denatured with a sample buffer (70 mM Tris-HCl, pH 6.5, 2.4% sodium dodecyl sulfate (SDS), 0.67 M urea, and 4 mM EDTA) at 60 °C for 20 minutes. The denatured VWF was then fractionated using 1% (w/v) SeaKem HGT agarose gel (Cambrex) and 5% SDS polyacrylamide gel electrophoresis (PAGE). The proteins were transferred onto a nitrocellulose membrane, blocked with 5% nonfat milk in Tris-buffered saline with Tween 20 (TBST) buffer (20 mM Tris-HCl, pH 7.5, 105 mM NaCl, and 0.1% Tween20), and incubated with anti-VWF IgG (0.2 μg/mL; Dako North America) overnight. After 3 washes with TBST, the bound primary antibody was detected using an IRDye 800CW-labeled goat antirabbit IgG (0.1 μg/mL; LI-COR) in TBST. The fluorescent signal was detected using the Odyssey imaging system (LI-COR) [34].

### 2.5 | Shear-dependent VWF cleavage by vortex

Purified plasma-derived VWF (30 nM) was incubated with a purified recombinant ADAMTS-13 enzyme (WT or R1206K; 50 nM) in a reaction buffer containing 20 mM HEPES, 150 mM NaCl, 5 mM CaCl_2_, and 0.5 mg/mL BSA (pH 7.5). The reaction mixture (final volume, 20 μL) was placed in 0.2-mL thin-walled polymerase chain reaction tubes (Fisher Scientific) and subjected to constant vortexing at 2500 rpm at 25 °C for 15 minutes or indicated time points to induce high turbulent shear [34]. The reaction was quenched by addition of an equal volume of sample buffer containing 125 mM Tris, 10% glycerol, 2% SDS, and 0.01% bromophenol blue (pH 6.8), followed by heating at 100 °C for 5 minutes. Cleavage products were resolved by 5% SDS-PAGE and transferred to nitrocellulose membranes. The membranes were blocked and incubated with a rabbit anti-VWF IgG (Dako), followed by an IRDye 800CW-labeled goat antirabbit IgG (LI-COR). The fluorescent signal of the specific cleavage product (molecular weight, ~350 kDa) was quantified using an Odyssey Infrared Imaging System (LI-COR).

### 2.6 | Microfluidic assay for thrombus formation

BioFlux microfluidic channels (Fluxion Bioscience) were coated with a fibrillar type I collagen (100 μg/mL; Chrono-Log) in 0.01 N of hydrochloric acid underflow (500/s) for 10 minutes. The surface was blocked with 0.5% BSA in phosphate-buffered saline (PBS). The platelets were labeled with fluorescein isothiocyanate–conjugated antihuman (1:10) or antimurine (1:100) CD41 antibody for 15 minutes and then added into anticoagulated whole blood samples obtained from *Adamts13*^*−/−*^ mice [35]. The whole blood was anticoagulated with a thrombin inhibitor, D-phenylalanyl-L-prolyl-L-arginine chloromethyl ketone (PPAC), with 0.5 μg/mL of WT-ADAMTS-13 or R1206K perfused under a high shear (100 dyne/cm^2^) over the collagen-coated surfaces for 3 minutes. Human recombinant ADAMTS-13 could effectively cleave murine VWF under these conditions as shown in our previous study [25,26]. The time-lapse digital images were collected every second for a total of 1 minute. The relative fluorescent intensity or platelet coverage was determined offline using Montage software, and data were analyzed using GraphPad Prism software. For high-resolution imaging, unbound platelets, leukocytes, and red blood cells were washed off from the channels with PBS for 10 minutes. The platelets adhered to the collagen surface were then fixed with ice-cold paraformaldehyde (4%) in PBS for 10 minutes. After being extensively washed and blocked with 2% BSA in PBS, the platelets were incubated with a fluorescein isothiocyanate–conjugated monoclonal anti-CD41. Images were obtained with Nikon A1 confocal fluorescence microscope.

### 2.7 | Inhibition of recombinant ADAMTS-13 activity by anti–ADAMTS-13 IgGs

The inhibitory potency (IC_50_) of a human monoclonal antibody (IgG4 4–16 or IgG4 4–20) against WT-ADAMTS-13 and R1206K variant was determined using the FRETS-VWF73 assay [8,28]. Recombinant enzyme (10 nM) was preincubated with a serially diluted anti–ADAMTS-13 IgG in a 5 mM Bis-Tris buffer (pH 6.0 or pH 7.0) containing 25 mM CaCl_2_, 0.005% Tween-20, and 1 mM Pefabloc. After 20 minutes of incubation at 25 °C, the residual ADAMTS-13 activity was detected by the cleavage of FRETS-VWF73 substrate (2 μM). Fluorescence generation (Ex340 nm/Em450 nm) was monitored in real time using a SpectraMax plate reader, and the initial rates were obtained from the linear portion of trace. The activity of each enzyme in the presence of IgG antibody was normalized to that in the absence of antibody (designated as 100% activity). Inhibition curves were fitted using a 4-parameter logistic model using GraphPad Prism 9.0, and IC_50_ values were derived from at least 3 independent experiments.

### 2.8 | Inhibition of recombinant ADAMTS-13 by patient plasma

Purified recombinant WT-ADAMTS-13 or R1206K (5 nM) was preincubated with 4 μL of citrated patient plasma in a buffer containing 5 mM Bis-Tris, pH 6.0, 25 mM CaCl_2_, 0.005% Tween-20, and 1 mM Pefabloc. After incubation at 37 °C for 1 hour, a FRETS-VWF73 substrate (2.0 μM) was added, and fluorescence generation was measured at 37 °C every 2 minutes for 1 hour in SpectraMax Paradigm (Molecular Devices) equipped with 485-nm excitation and 535-nm emission filters. The residual proteolytic activity was normalized to WT-ADAMTS-13 (defined as 100% activity).

### 2.9 | ADAMTS-13 conformation index

To minimize the structure perturbation, recombinant WT-ADAMTS-13 and R1206K proteins in the conditioned media were used. First, ADAMTS-13 antigen concentrations were determined using an ELISA as previously described [24,36–38]. This was done by capturing ADAMTS-13 proteins by a mouse monoclonal antimetalloprotease IgG (3H9) and detected by a biotinylated antibody targeting the eighth TSP1 repeat of ADAMTS-13. The binding of ADAMTS-13 proteins to a cryptic antibody (IC4) and the relative conformational index of recombinant ADAMTS-13 proteins in the absence or presence of various autoantibodies were assessed using the method described previously [24,36–38].

### 2.10 | Western blotting and immunoprecipitation

Stably transfected human embryonic kidney-293 cells expressing WT-ADAMTS-13 and R1206K variant were lysed with a lysis buffer (20 mM HEPES, pH 7.9, 150 mM NaCl, 1mM MgCl_2_, 1% NP40, 10 mM NaF, 0.2 mM NaVO_4_, 10 mM β-glycerol phosphate, and proteinase inhibitors; Roche). The lysates were cleared by centrifugation and used for western blotting or immunoprecipitation. The proteins were resolved by 10% SDS-PAGE and transferred to a polyvinylidene fluoride membrane for blotting using an immobilon western chemiluminescent horseradish peroxidase substrate (Millipore). Anti-PRMT1 antibody (MilliporeSigma), anti-His antibody (Abcam), antiasymmetric dimethyl arginine IgG (ThermoFisher), anti–His IgG bead (Invitrogen), and β-actin murine monoclonal antibody (Sigma-Aldrich) were all commercially available. Rabbit anti–ADAMTS-13 IgGs were produced in house as previously described [39].

## 3 | RESULTS

### 3.1 | R1206K variant exhibited an enhanced activity toward VWF73 substrate

**FIGURE 1:**
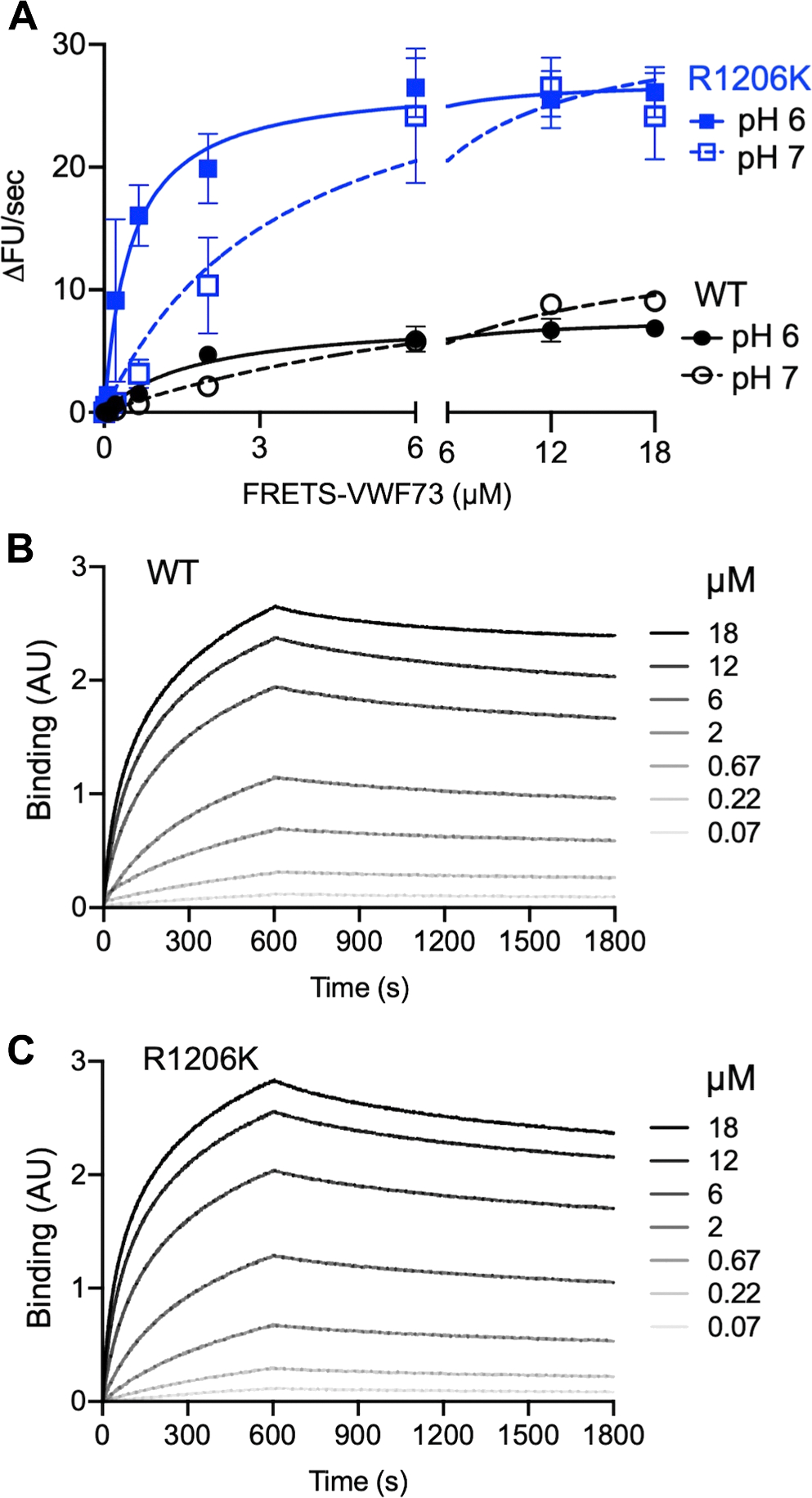
Kinetic assessment of recombinant ADAMTS-13 interaction with its substrates. (A) Kinetic cleavage of FRETS-VWF73 (0–18 μM) by wild-type (WT)-ADAMTS-13 and R1206K at pH 6.0 (solid lines) and pH 7.0 (dashed lines), respectively. The *V*_max_, *K*_m_, and catalytic efficiency (*V*_max_*/K*_m_) were determined (Table 1). (B, C) Kinetic binding interaction between WT or K1206K and its uncleavable substrate VWF73^L1603A^ (0–18 μM) using a biolayer interferometry assay. The association rate (*k*_a_), dissociation rate (*k*_dis_), and overall dissociation constant (*K*_d_) were determined using the Octet BLl system software (Table 2).

To quantitatively assess the proteolytic activity of recombinant ADAMTS-13 R1206K variant, we performed the FRETS-based assay under 2 different pH conditions (pH 6.0 and pH 7.0). Low pH has been shown to activate plasma native ADAMTS-13 [40,41]. This assay enables real-time monitoring of VWF peptide cleavage as a function of substrate concentration (Figure 1A). Michaelis–Menten constant (*K*_m_), the maximum reaction velocity (*V*_max_), and the catalytic efficiency (*K*_cat_*/K*_m_) could be obtained for both WT-ADAMTS-13 and R1206K. As shown in Table 1, at the pH 6.0 condition, the R1206K variant exhibited a significantly higher *V*_max_ (mean ± SEM, 27.1 ± 1.6 RFU/min) than WT-ADAMTS-13 (7.8 ± 0.8 RFU/min), and a significantly lower *K*_m_ value (mean ± SEM, 0.5 ± 0.2 μM) than WT-ADAMTS-13 (1.8 ± 0.7 μM). Additionally, the catalytic efficiency for R1206K was 16.5, ~4 times greater than that of WT-ADAMTS-13 (4.4). At the pH 7 condition, both WT-ADAMTS-13 and R1206K exhibited increased *V*_max_ values, yet the *K*_cat_ for R1206K (mean ± SEM, 32.3 ± 5.4 RFU/min) remained 2 times higher than that for WT-ADAMTS-13 (14.6 ± 2.1 RFU/min). While the *K*_m_ values for both were also increased at neutral pH, the mean catalytic efficiency for R1206K (9.4) was still significantly higher than that of WT-ADAMTS-13 (1.5). These findings demonstrate the gain-of-function property of the R1206K variant regardless of the assay conditions being used.

### 3.2 | R1206K exhibited an increased binding affinity with its uncleavable VWF73 substrate

To gain insight into the molecular mechanism underlying gain-of-function of R1206K variant, we determined the binding kinetic between R1206K and its surrogate substrate VWF73 ^L1603A^ using the BLI assay. The VWF73^L1603A^ peptide is not cleavable by ADAMTS-13 as described previously [31]. The association rate constant (*k*_on_), the dissociation rate constant (*k*_dis_), and the equilibrium dissociation constant (*K*_d_), were determined for binding between WT-ADAMTS-13 and VWF73^L1603A^ (Figure 1B) or between R1206K and VWF73^L1603A^ (Figure 1C). All kinetic results are shown in Table 2. Both WT-ADAMTS-13 and R1206K exhibited a similar binding affinity toward VWF73^L1603A^, with the dissociation constant (*K*_d_) for WT-ADAMTS-13 and R1206K of 4.8 × 10^−6^ M and 4.6 × 10^−6^ M, respectively. However, the R1206K variant showed a slightly higher association rate (*k*_a_ = 5.3 × 10^−3^/s) than WT-ADAMTS-13 (*k*_a_ = 4.9 × 10^−3^/s), but similar dissociation rate (2.3 × 10^−3^/s vs 2.2 × 10^−3^/s). The *R*^2^ values of 0.99 for both variants suggest the robustness of the fitting and reliability of the measurements. These results indicate that the gain-of-function of R1206K variant may not be the result of increased substrate binding.

### 3.3 | R1206K variant exhibited more activity than WT-ADAMTS-13 for cleaving multimeric VWF under denaturing conditions

**FIGURE 2:**
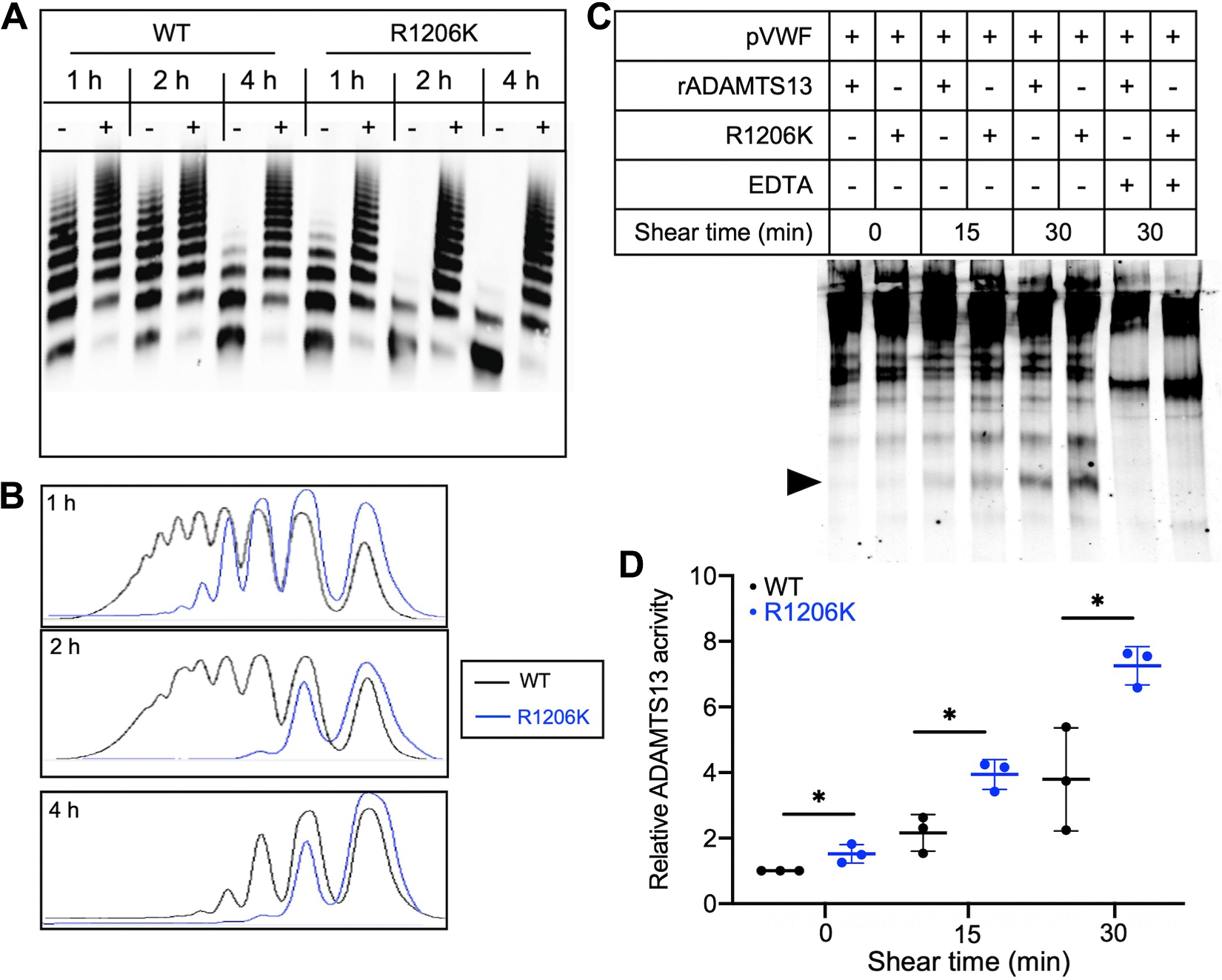
Proteolytic cleavage of multimeric von Willebrand factor (VWF) by wild-type (WT)-ADAMTS-13 and R1206K under denaturing conditions. (A) Western blotting multimer analysis demonstrates the proteolytic cleavage of full-length multimeric VWF by WT-ADAMTS-13 and R1206K following 1, 2, and 4 hours of incubation on a dialysis membrane floating on top of a buffer containing 5 mM Tris-HCl, pH 8.0 and 1.0 M urea in the absence (−) or presence (+) of 10 mM EDTA. (B) Representative densitometric scanning demonstrates the intensity of various VWF multimer bands from high-molecular-weight (HMW) to low-molecular-weight (LMW) multimers after being cleaved by WT-ADAMTS-13 (black line) and R1206K (blue line). (C) Representative multimer analysis of purified human VWF incubated with recombinant WT-ADAMTS-13 or the R1206K variant under constant shear stress (vortexing) for 0, 15, and 30 minutes. The arrowhead indicates the specific VWF cleavage product generated by ADAMTS-13. Samples containing EDTA served as negative controls for enzymatic activity. (D) Densitometric quantification of the VWF cleavage product at the indicated time points. Data represents the relative quantification level normalized to baseline.

To confirm the gain-of-function of R1206K variant, we performed the cleavage of multimeric VWF by WT-ADAMTS-13 vs R1206K under denaturing conditions. As shown, in the presence of WT-ADAMTS-13 or R1206K without EDTA, purified recombinant human VWF multimers were cleaved in a time-dependent manner (Figure 2A). Densitometric analysis demonstrated the loss of high-molecular-weight VWF multimers within 1 hour of incubation with R1206K, which was not the case even after 2 hours of incubation with WT-ADAMTS-13 (Figure 2B).

### 3.4 | R1206K exhibited more activity than WT-ADAMTS-13 for cleavage of multimeric VWF under mechanical shear

To demonstrate the physiological relevance, we also performed the proteolytic cleavage of multimeric VWF under mechanical shear. Purified human VWF was incubated with WT-ADAMTS-13 or R1206K and subjected to constant vortexing for 0, 15, and 30 minutes. SDS-PAGE and western blotting analysis demonstrated the time-dependent increase of proteolytic cleavage product (350 kD) with both WT-ADAMTS-13 and R1206K. No cleavage band was detected in the reaction with EDTA, confirming that the cleavage product is specific (Figure 2C). Quantitation of the 350-KD band indicated the higher activity of R1206K than WT-ADAMTS-13 for proteolytic cleavage of multimeric VWF under mechanical shear (Figure 2D).

### 3.5 | R1206K is more potent than WT-ADAMTS-13 for antithrombosis under flow

**FIGURE 3:**
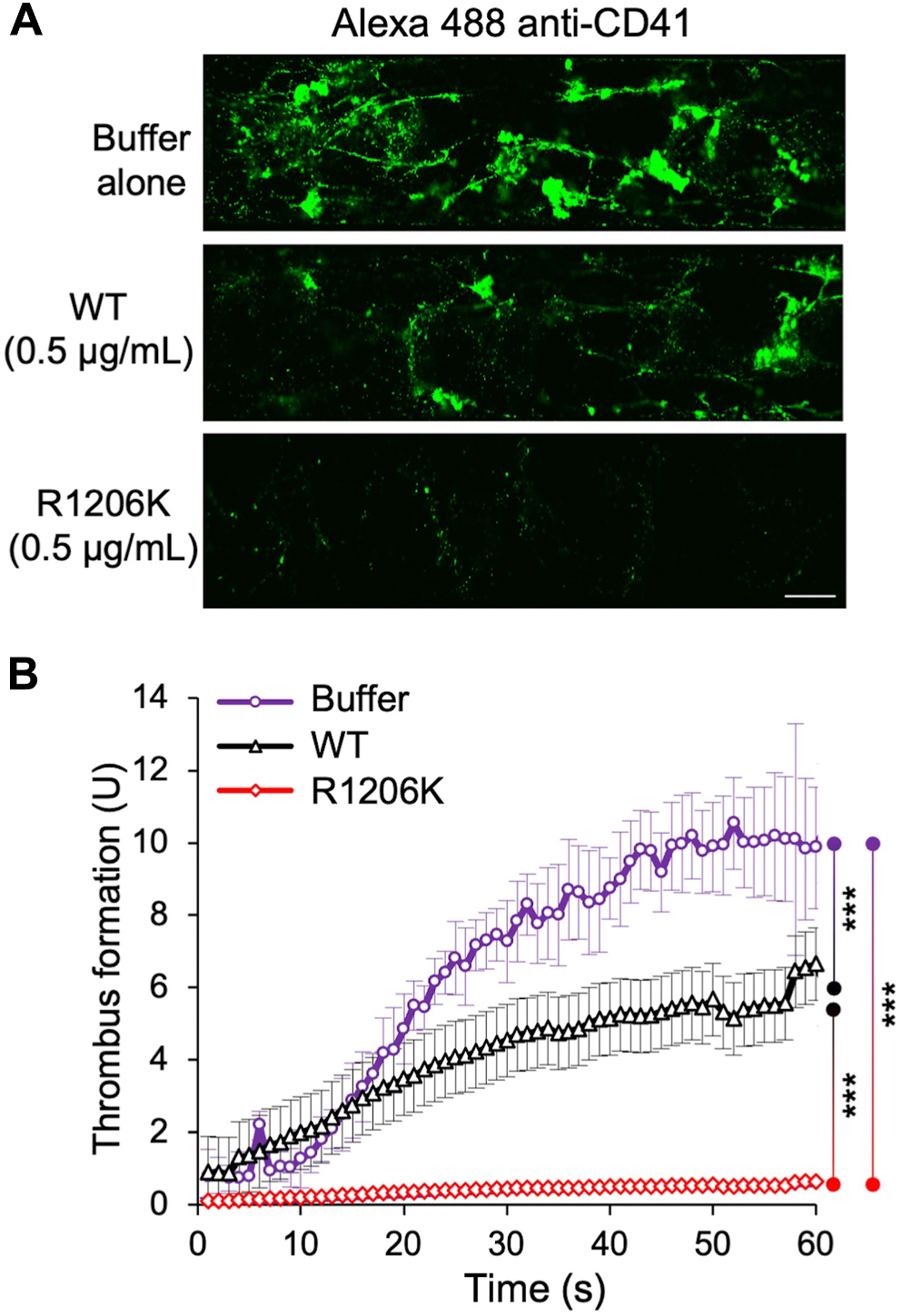
Microfluidic shear-based assay for assessing anti-thrombotic activity of wild-type (WT)-ADAMTS-13 and R1206K. (A) Representative images showing the final surface coverage of fluorescent anti-CD41 positive platelets in the absence (top, buffer alone) and the presence (0.5 μg/mL) of WT-ADAMTS-13 (middle) and R1206K (bottom) under arterial shear (100 dyne/cm^2^). Scale bar, 20 μm. (B) The rates of thrombus formation (means ± SEM) as a function of time were obtained from 3 independent experiments. Krustal–Wallis U-test was performed to compare the difference in the rates of thrombus formation among 3 groups. ***P < .005.

To further assess the gain-of-function activity of R1206K under more physiological conditions, we used a BioFlux microfluidic assay. Following the perfusion of a PPACK (thrombin inhibitor) anti-coagulated whole blood from *Adamts13*^−*/*−^ mice in the presence of WT-ADAMTS-13 or R1206K variant (at a final concentration of 0.5 μg/mL), the final coverage (Figure 3A) and the rate (Figure 3B) of accumulation of fluorescein-labeled platelets on a fibrillar collagen coated surface were more dramatically reduced with R1206K than WT-ADAMTS-13 (*n* = 3; *P* < .005). These results further confirm that the R1206K variant has an enhanced proteolytic activity toward newly released ultralarge VWF multimers under physiologically relevant conditions.

### 3.6 | R1206K adopted a more open conformation than WT-ADAMTS-13

**FIGURE 4:**
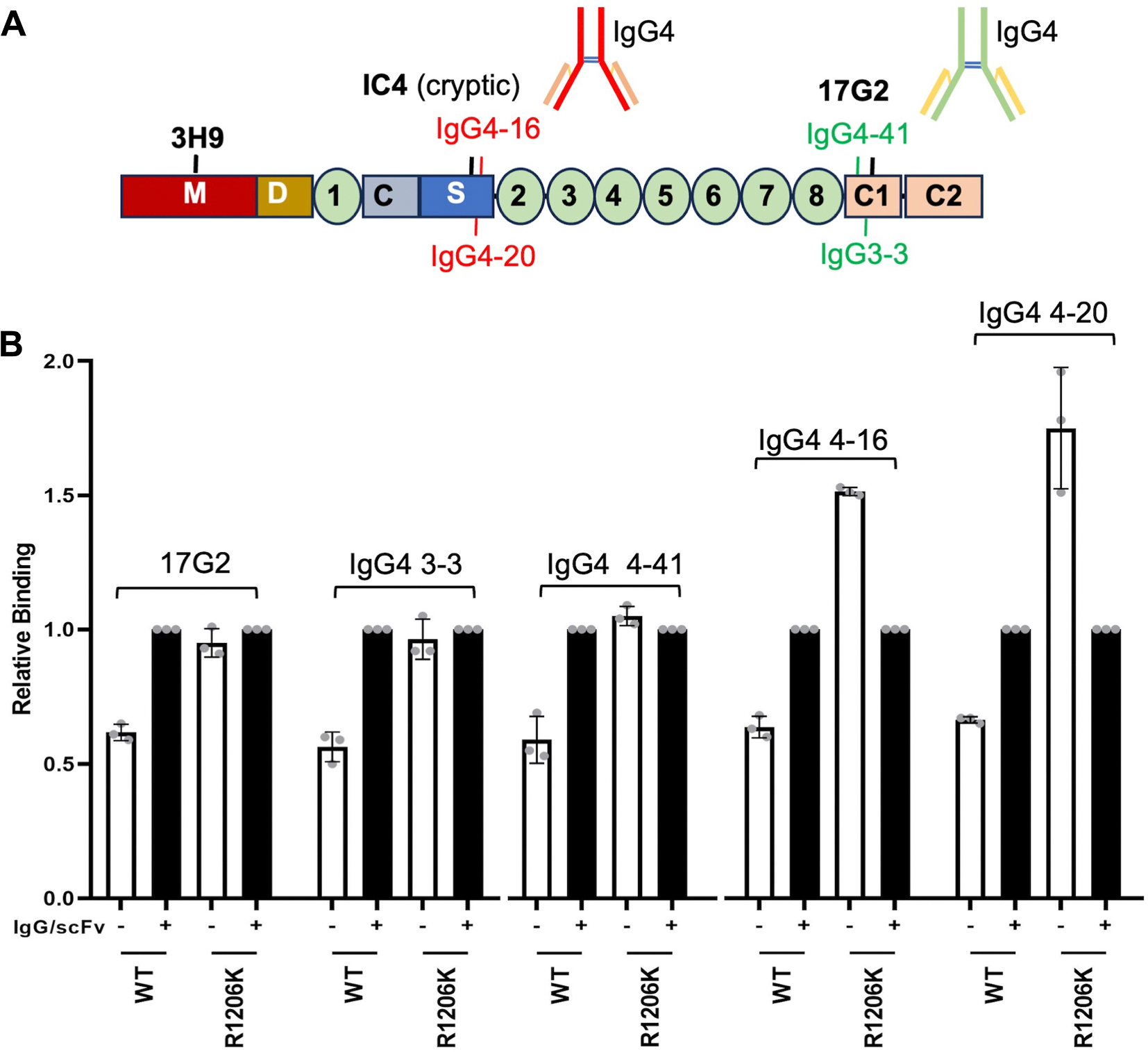
Conformational index for wild-type (WT)-ADAMTS-13 and R1206K upon antibody binding. (A) Schematic representation of a full-length ADAMTS-13 protein with various antibody-binding sites shown. Those that bind the spacer (IgG4 4–16 and IgG4 4–20) are inhibitory antibodies (red) and those that bind the distal CUB1 (IgG4 3–3 and IgG4 4–41) are activating antibodies (green) all isolated from patients with immune-mediated thrombotic thrombocytopenic purpura (iTTP). 3H9 and IC4 are murine monoclonal antibodies used to determine open conformational ADAMTS-13 (bold). (B) Relative binding of WT-ADAMTS-13 and R1206K to antibody recognizing the cryptic epitope in the spacer domain (IC4) following incubation with anti-CUB1 17G2 or various human monoclonal antibodies isolated from patients with iTTP.

Native ADAMTS-13 under physiological conditions typically exists in a closed or latent conformation, which can be activated by binding to a VWF ligand or an antibody against the distal domain of ADAMTS-13 [28,41]. We took advantage of a monoclonal antibody (IC4) that only recognizes the cryptic epitope in the spacer domain of ADAMTS-13 to probe the conformational difference between WT-ADAMTS-13 and R1206K. Recombinant ADAMTS-13 proteins were captured by antimetalloprotease IgG (3H9) in the absence or presence of activating antibodies that target the distal CUB domains (17G2, IgG4 3–3, and IgG4 4–41) or inhibitory antibodies that recognize the spacer domain of ADAMTS-13 (IgG4 4–16 and IgG4 4–20) (Figure 4A). Anti-CUB monoclonal antibodies (17G2, IgG4 3–3, and IgG4 4–41) could all increase the binding of IC4 (cryptic) to WT-ADAMTS-13 but not R1206K; similarly, monoclonal antispacer antibodies (IgG4 4–16 and IgG4 4–20) could also increase the binding of IC4 to WT-ADAMTS-13 but paradoxically reduced the binding of IC4 to R1206K variant (Figure 4B) under the same conditions. These results suggest that R1206K exhibits a different but more open conformation than WT-ADAMTS-13, which may explain the functional differences.

### 3.7 | R1206K displays a modestly reduced susceptibility to inhibition by both monoclonal antibodies and patient-derived autoantibodies

**FIGURE 5:**
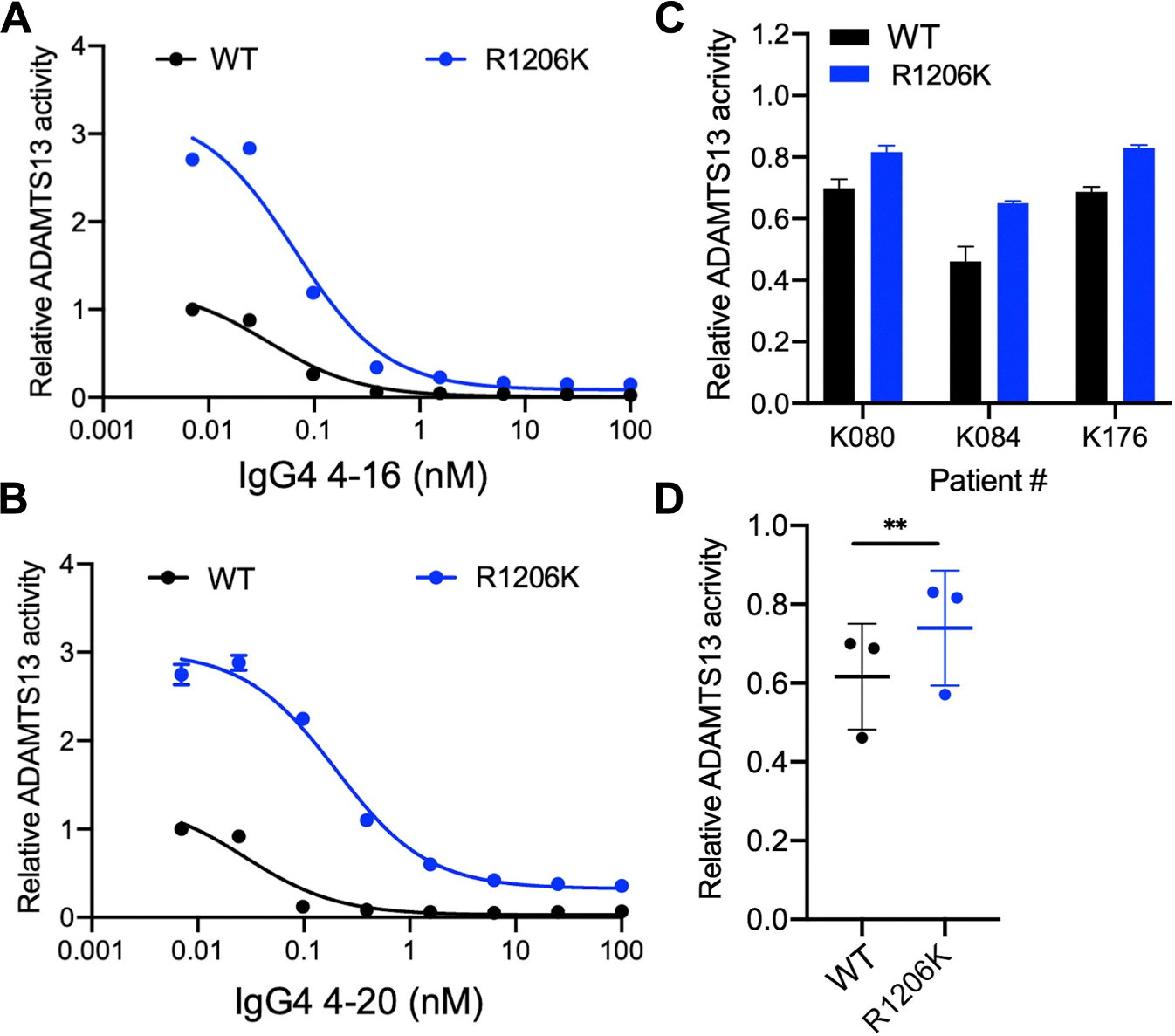
Inhibition of wild-type (WT)-ADAMTS-13 and R1206K by autoantibodies from immune-mediated thrombotic thrombocytopenic purpura (iTTP). Dose-dependent inhibition of cleavage activity of WT-ADAMTS-13 and R1206K (2 μg/mL) by human monoclonal anti-ADAMTS-13 antibodies IgG4 4–16 (A) and IgG4 4–20 (B), respectively. The half-maximal inhibitory concentration (IC_50_) of each inhibitor was determined using a nonlinear regression fitting (Table 3). (C) Residual proteolytic activity of recombinant WT-ADAMTS-13 (black bars) and R1206K (blue bars) following incubation with patient plasma. (D) Data are normalized to the activity of the respective enzyme in the absence of inhibitor (*P* < .01, Student’s *t*-test).

To determine whether R1206K is more resistant to autoantibody inhibition, we performed the inhibition titration by increasing concentration of an inhibitory antibody. As shown, human monoclonal IgG4 4–16 (Figure 5A) and IgG4 4–20 (Figure 5B) inhibited both WT-ADAMTS-13 and R1206K in a concentration-dependent manner. Kinetic data shown in Table 3 demonstrated that the IC_50_ for inhibiting WT-ADAMTS-13 and R1206K by IgG4 4–20 was 0.03 and 0.20 nM, respectively. The IC_50_ for inhibiting WT-ADAMTS-13 and R1206K by IgG4 4–16 was 0.04 and 0.07 nM, respectively. These results indicate that R1206K has a modestly reduced susceptibility to inhibition by some but not all autoantibodies from iTTP. Interestingly, in the presence of anti-CUB scFv3–3, the inhibitory effect of IgG4 4–16 on WT-ADAMTS-13 was significantly enhanced (IC_50_, 0.3 ± 0.2 nM), while no change (IC_50_, 1.2 ± 1.4 nM) was observed for R1206K inhibition by IgG4 4–16 in the presence of scFv3–3 (Supplementary Figure S1 and Table S1). These results suggest that R1206K is in a different conformation that may confer some resistance to inhibitory antibodies. We further validated the partial resistance to autoantibodies using plasma samples from patients with iTTP. Functional assays with samples from 3 patients (K080, K084, and K176) who had severe ADAMTS-13 deficiency (<5% activity) and high inhibitor titers (23–144 Bethesda Units) (Table 4) demonstrated that R1206K consistently displayed more partial resistance to inhibition than WT-ADAMTS-13 (Figure 5C, D).

### 3.8 | R1206K exhibited a reduced binding to IgG autoantibodies

**FIGURE 6:**
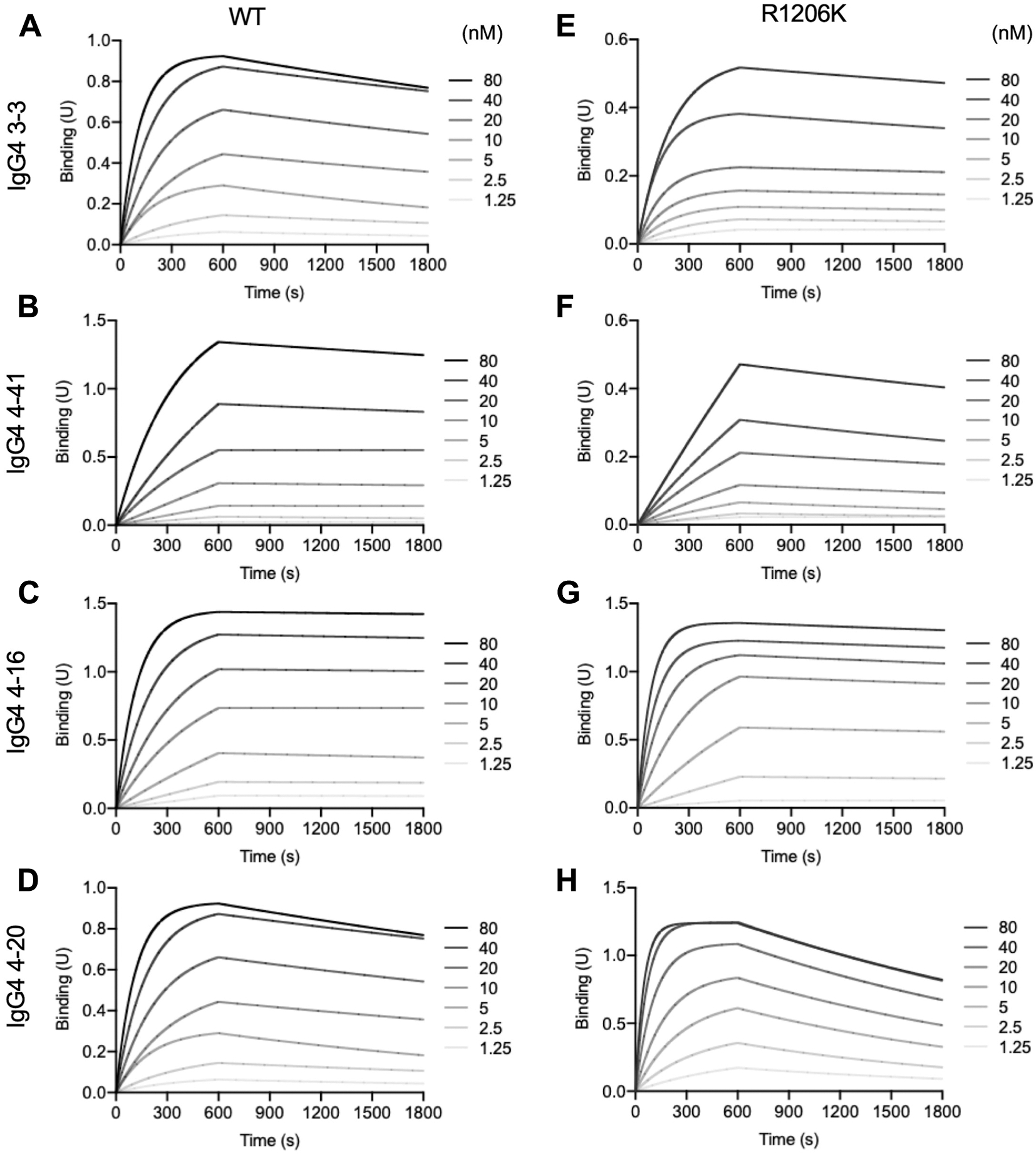
Kinetic binding interaction between R1206K (or WT-ADAMTS-13) and human monoclonal antibodies. Binding kinetics showing an association and a dissociation of WT-ADAMTS-13 (A–D) and R1206K (E–H) interacting with IgG4 3–3 (A, E), IgG4 4–41 (B, F), IgG4 4–16 (C, G), and IgG4 4–20 (D, H). *K*_d_, *k*_a_, and *k*_dis_ were determined using a global 1:1 fit (Table 5).

To determine the antibody-binding affinity, we performed the BLI assay. As shown in Figure 6, both WT-ADAMTS-13 and R1206K bound both activating and inhibitory antibodies in a concentration-dependent manner. When compared with WT-ADAMTS-13 binding (*K*_d_, 0.50 ± 0.03 nM), the R1206K variant showed a slightly reduced binding to IgG4 4–20 (*K*_d_, 0.80 ± 0.01 nM). This may have primarily resulted from an accelerated dissociation rate (*k*_dis_, 1.3 × 10^−4^) for R1206K compared with that (*k*_dis_, 5.2 × 10^−4^ 1/s) for WT-ADAMTS-13. A modest reduction in affinity was also observed for binding of R1206K to inhibitory IgG4 4–16 (*K*_d_, 0.70 ± 0.02 nM), compared with binding of WT-ADAMTS-13 to IgG4 4–16 (*K*_d_, 0.40 ± 0.02 nM). In contrast, the R1206K variant demonstrated a markedly increased affinity toward IgG3–3 (*K*_d_, 0.06 ± 0.002 nM) compared with WT-ADAMTS-13 (*K*_d_, 0.30 ± 0.046 nM) due to its accelerated association (8.8 × 10^5^ to 1.4 × 10^6^ 1/Ms) and reduced dissociation (2.6 × 10^−4^ to 7.8 × 10^−5^ 1/s). Conversely, binding to IgG4 4–41 was substantially weakened (*K*_d_, 0.80 ± 0.03 to 2.7 ± 0.2 nM), driven by a ~28-fold increase in dissociation (1.1 × 10^−5^ to 3.0 × 10^−4^ 1/s) (Table 5).

Together, these findings suggest that R1206K is in a different and more open conformation than WT-ADAMTS-13, exhibits an enhanced proteolytic activity toward VWF under various conditions and appears to show a reduced binding and susceptibility to autoantibody inhibition.

## 4 | DISCUSSION

In this study, we demonstrate that R1206K is a gain-of-function ADAMTS-13 variant, with 4- to 5-fold increase of specific proteolytic activity toward a surrogate VWF73 substrate. R1206K variant also exhibits a significantly increased activity for proteolytic cleavage of multimeric VWF under denaturing conditions and under mechanical shear. More important, R1206K exhibits a dramatically increased efficacy inhibiting platelet-rich thrombosis under arterial flow. Furthermore, R1206K appears to exhibit a different but more open conformation than WT-ADAMTS-13 and is at least partially resistant to inhibition by autoantibodies from iTTP. These findings highlight the crucial role of C-terminus–mediated autoinhibition or arginine methylation in regulation of ADAMTS-13 function and autoantibody susceptibility.

Previous studies have demonstrated that ADAMTS-13 undergoes various posttranslational modifications including N- and O-linked glycosylation [42,43] and arginine methylation [24], which is important for its proteolytic activity and antibody binding. The arginine (R) at position of 1206 in the CUB1 domain appears to be highly conserved among ADAMTS-13 species from zebrafish to mammals [24]. This arginine on WT-ADAMTS-13 is usually methylated, which may be important for interaction with the proximal-terminal spacer or other adjacent domains to keep ADAMTS-13 in so-called a closed conformation and to maintain its latency [15]. When this arginine is changed to lysine (K), it eliminates the methylation at 1206, which may disrupt the interaction between the CUB1 and spacer or other adjacent domains, resulting in a different but more open conformation. This hypothesis is demonstrated by the conformational index analysis where R1206K binds more to the cryptic antibody than WT-ADAMTS-13. These results are consistent with our previous data, demonstrating a significant increase of proteolytic activity of WT-ADAMTS-13, but not R1206K toward FRETS-VWF73 upon lowering pH from 7.0 to 6.0 or following addition of an anti-CUB antibody, scFv3–3 [24].

The precise mechanism by which R1206K exhibits a reduced susceptibility to autoantibodies remains to be fully elucidated, although our data suggest it may relate to its conformation or posttranslational arginine methylation. As indicated by the kinetic and binding analyses, R1206K stabilizes the enzyme in a constitutively open conformation. This global structural shift likely alters the accessibility or geometry of the conformational epitopes targeted by substrates and pathogenic antibodies. Previous epitope mapping studies using several inhibitory monoclonal antibodies (eg, scFv4–16 and scFv4–20) have demonstrated that these inhibitors recognize a complex surface epitope formed by multiple discontinuous loops; therefore, the conformational rearrangement induced by R to K mutation may perturb the spatial alignment of these loops, thus reducing antibody affinity. Consequently, this gain-of-function variant, with its dual profile of enhanced enzymatic activity and reduced sensitivity to inhibition, may represent a novel promising candidate for the development of next-generation therapies for iTTP, which is currently managed by a triple therapy, including TPE, caplacizumab, and immunosuppression (eg, corticosteroids and rituximab) [12,44]. The gain-of-function R1206K variant can certainly be used for treatment of hTTP and perhaps iTTP with a much reduced dose.

We conclude that the R1206K variant exhibits a different but more open conformation than WT-ADAMTS-13 and shows an enhanced enzymatic activity toward both peptidyl and multimeric VWF substrates under various conditions. Additionally, R1206K demonstrates a dramatically increased antithrombotic activity compared with WT-ADAMTS-13 under arterial flow. Interestingly, the R1206 variant also exhibits a modestly reduced susceptibility to anti-ADAMTS-13 autoantibody inhibition. Together, these combined properties of R1206K make it a promising candidate for the development of next-generation therapeutics for TTP and other potential inflammatory thrombotic disorders.

## Supplementary Material

Suppl. Figure S1

The online version contains supplementary material available at https://doi.org/10.1016/j.jtha.2026.03.010.

## Figures and Tables

**TABLE 1:** Kinetic parameters of cleaving FRETS-VWF73 by WT-ADAMTS-13 and R1206K at various pH conditions.

pH	Parameters	WT	R1206K
6.0	*V*_max_ (RFU/min)	7.8 ± 0.8	27.1 ± 1.6
	*K*_m_ (μM)	1.8 ± 0.7	0.5 ± 0.2
	*R* ^2^	0.96	0.93
	Catalytic efficiency	4.4	16.5
7.0	*V*_max_ (RFU/min)	14.6 ± 2.1	32.3 ± 5.4
	*K*_m_ (μM)	9.5 ± 3.0	3.5 ± 1.9
	*R* ^2^	0.98	0.93
	Catalytic efficiency	1.5	9.4

FRETS, Fluorescent energy transfers; *K*_m_, Michaelis–Menten constant; R1206K, ADAMTS-13 variant with arginine to lysine change at position of 1206; RFU/min, relative fluorescence unit per minute; *V*_max_, maximal velocity; WT, wild type.

**TABLE 2:** Binding affinity between recombinant ADAMTS-13 proteins and its substrate FRETS-VWF73^L1603A^.

Constructs	*K*_d_ (M)	*k*_obs_ (1/s)	*k*_dis_ (1/s)
WT (*R*^2^ = 0.99)	4.8 × 10^−6^	4.9 × 10^−3^ ± 3.9 × 10^−5^	2.2 × 10^−3^ ± 8.2 × 10^−6^
R1206K (*R*^2^ = 0.99)	4.6 × 10^−6^	5.3 × 10^−3^ ± 4.5 × 10^−5^	2.3 × 10^−3^ ± 1.9 × 10^−5^

*K*_d_, dissociation constant; *k*_dis_, dissociation rate constant; *k*_obs_, observed association constant; R1206K, ADAMTS-13 variant with arginine to lysine change at position of 1206; WT, wild-type.

**TABLE 3:** IC50 for inhibition of WT-ADAMTS-13 and R1206K by anti-ADAMTS-13 IgG4s.

IC50 (nM)	WT	R1206K	Fold change
IgG4 4–20 (*R*^2^)	0.03 (0.93)	0.20 (0.99)	7.3
IgG4 4–16 (*R*^2^)	0.04 (0.97)	0.07 (0.97)	1.8

IC_50_, the concentration of an inhibitor required to reduce enzyme activity by 50%; IgG, immunoglobulin; R1206K, ADAMTS-13 variant with arginine to lysine change at position of 1206; WT, wild type.

**TABLE 4:** Clinical characteristics of the iTTP patient plasma samples used in the inhibition assays.

Sample No.	ADAMTS-13 activity (%)	Anti–ADAMTS-13 IgG (IU/mL)
K080	3	144.0
K084	0	71.0
K176	1	23.3

IgG, immunoglobulin; iTTP, immune-mediated thrombotic thrombocytopenic purpura.

**TABLE 5:** Kinetic binding of anti–ADAMTS-13 IgG4s to WT rADAMTS-13 and R1206K variant.

IgG	WT				R1206K				*P*		
	*K*_d_ (nM)	*k*_a_ (1/Ms)	*k*_dis_ (1/s)	*R* ^2^	*K*_d_ (nM)	*k*_a_ (1/Ms)	*k*_dis_ (1/s)	*R* ^2^	*K*_d_ (nM)	*k*_a_ (1/Ms)	*k*_dis_ (1/s)
IgG4 3-3	0.3 ± 0.05	8.8 ± 0.1 × 10^5^	2.6 ± 0.03 × 10^−4^	0.98	0.06 ± 0.002	1.4 ± 0.02 × 10^6^	7.8 ± 0.3 × 10^−5^	0.97	< 1 × 10^−7^	< 1 × 10^−7^	< 1 × 10^−8^
IgG4 4-41	0.8 ± 0.03	1.3 ± 0.38 × 10^4^	1.1 ± 0.17 × 10^−5^	0.99	2.7 ± 0.2	1.1 ± 0.08 × 10^5^	3.0 ± 0.04 × 10^−4^	0.97	< 1 × 10^−10^	< 1 × 10^−9^	< 1 × 10^−12^
IgG4 4-16	0.4 ± 0.017	7.0 ± 0.03 × 10^5^	2.7 ± 0.06 × 10^−5^	0.99	0.7 ± 0.02	8.0 ± 0.02 × 10^5^	5.3 ± 0.04 × 10^−5^	0.99	< 1 × 10^−12^	< 1 × 10^−11^	< 1 × 10^−12^
IgG4 4-20	0.5 ± 0.03	2.6 ± 0.08 × 10^5^	1.3 ± 0.01 × 10^−4^	0.99	0.8 ± 0.014	6.6 ± 0.1 × 10^5^	5.2 ± 0.04 × 10^−4^	0.96	< 1 × 10^−9^	< 1 × 10^−11^	< 1 × 10^−13^

All data were generated by 1:1 global curve fitting, with corresponding *R*^2^ values shown.

IgG, immunoglobulin G; *k*_a_, association rate constant; *K*_d_, equilibrium dissociation constant; *k*_dis_, dissociation rate constant; R1206K, ADAMTS-13 variant with arginine to lysine change at position of 1206; WT, wild type.

## Essentials

- Therapeutic option for TTP remains to be quite limited.
- Recombinant ADAMTS-13 R1206K displays an open conformation and is a gain-of-function enzyme toward von Willebrand factor.
- The R1206K variant also exhibits an enhanced activity. effecitively inhibiting thrombus formation under flow.
- This gain-of-function ADAMTS-13 may be explored for therapeutic implication in TTP.
