# Supplementary figures and images for: ADAMTS-13 R1206K variant exhibits an open conformation and an enhanced proteolytic activity toward von Willebrand factor
Source: J Thromb Haemost. Author manuscript; Available in PMC 2026 Jul 28. (PMC13411380; doi:10.1016/j.jtha.2026.03.010)

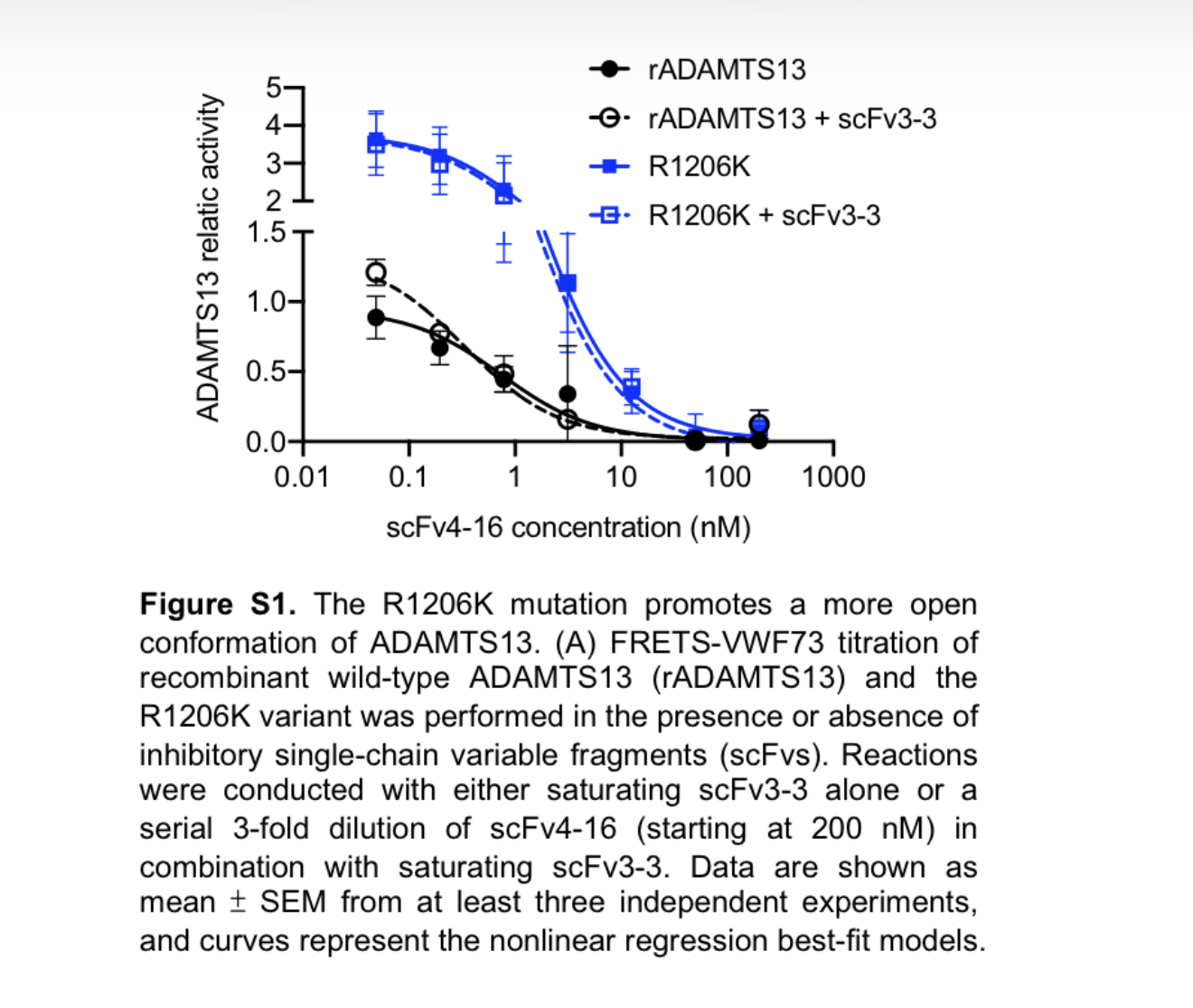

Supplement: Suppl. Figure S1 [file NIHMS2186532-supplement-Suppl__Figure_S1.png]
